# Immunometabolism drives metabolic and infectious disease progression through bidirectional metabolic reprogramming and epigenetic remodeling

**DOI:** 10.3389/fimmu.2026.1914506

**Published:** 2026-08-25

**Authors:** Wenbin Tan

**Affiliations:** School of Basic Medicine, Jining Medical University, Jining, Shandong, China

**Keywords:** epigenetic remodeling, immunometabolism, infection, inflammation, innate immune memory, metabolic diseases, metabolic reprogramming, trained immunity

## Abstract

Immunometabolism has become a central mechanism governing innate and adaptive immune responses. The field has moved from cataloguing metabolic shifts during immune activation to understanding how specific pathways control immune cell fate and function. This review brings together current knowledge on immunometabolic regulation in metabolic and infectious diseases, emphasizing bidirectional crosstalk between these domains. We address controversies over whether inflammation causes insulin resistance or follows from it, examine the distinct metabolic programs of different immune cell types, and explore trained immunity as a link between innate immune memory and metabolic disease. The review also covers pathogen-specific metabolic strategies and host countermeasures, surveys emerging immunometabolic therapies, and flags unresolved questions for future work. Among promising strategies, mechanistic target of rapamycin (mTOR) inhibitors and glucagon-like peptide-1 (GLP-1) receptor agonists have advanced to clinical trials for immunometabolic indications, whereas epigenetic reprogramming and nanoparticle-based metabolic delivery remain in preclinical development.

## 1 Introduction

**Figure 1:**
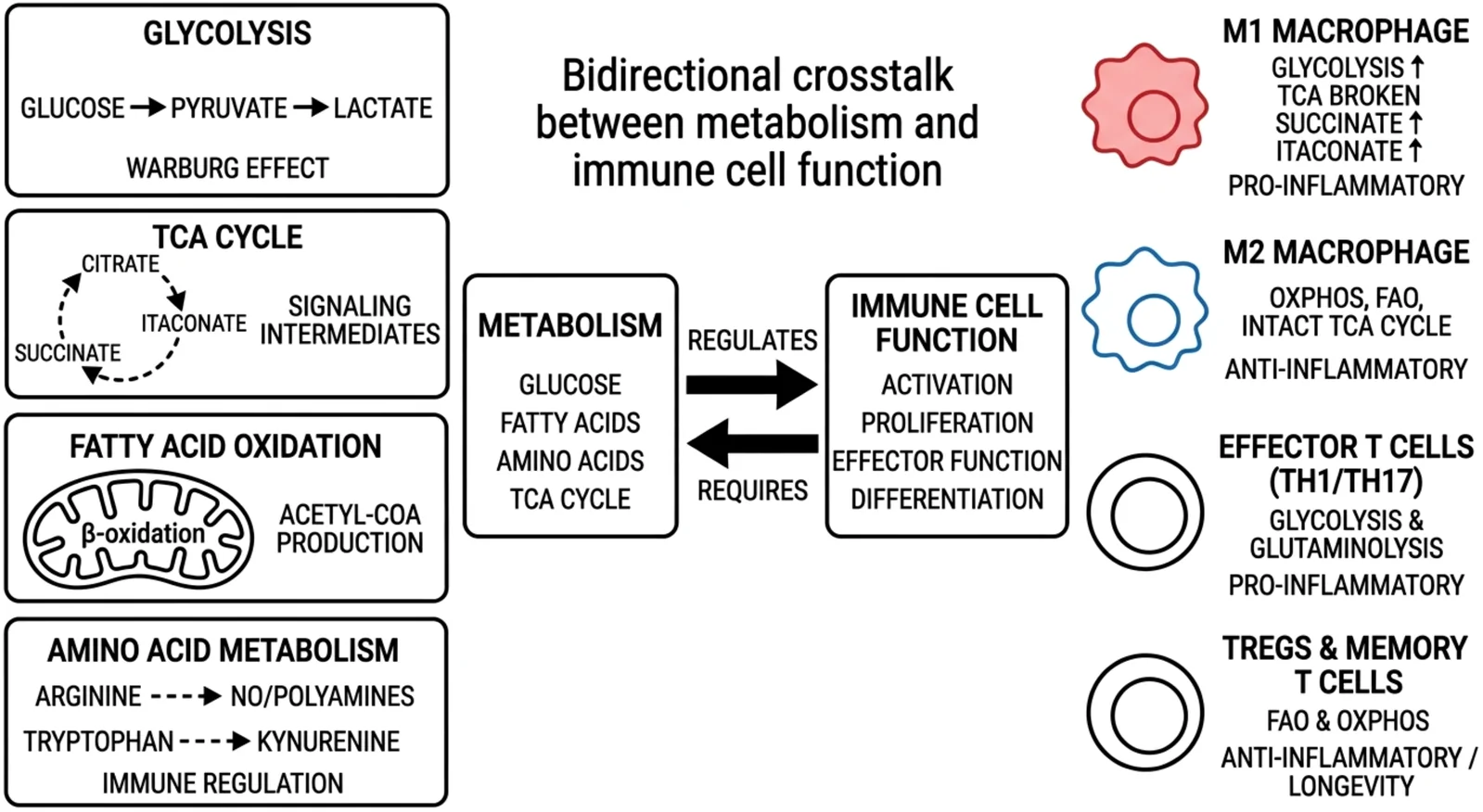
Overview of immune metabolism in health and disease. Schematic illustration of the bidirectional crosstalk between core metabolic pathways (glycolysis, TCA cycle, fatty acid oxidation, and amino acid metabolism) and immune cell function. Distinct metabolic profiles of M1 macrophages (glycolysis-driven, pro-inflammatory) versus M2 macrophages (OXPHOS-driven, anti-inflammatory), and effector T cells versus regulatory/memory T cells are highlighted. Created with BioRender.com.

The immune system and metabolic regulation are intimately connected through bidirectional crosstalk that has evolved to optimize host defense while maintaining metabolic homeostasis. Immune cell activation, proliferation, and effector functions demand substantial energy and biosynthetic precursors. Meeting these demands requires rapid metabolic reprogramming. Conversely, systemic and tissue-specific metabolic states profoundly influence the magnitude and quality of immune responses, creating feedback loops that can either protect against or contribute to disease pathogenesis (1, 2). This bidirectional crosstalk is summarized in **Figure 1**.

Immunology and metabolism were long studied as separate disciplines. Immunologists focused on signaling pathways and transcriptional regulation; metabolic researchers examined energy homeostasis and nutrient sensing. The realization that metabolic intermediates double as signaling molecules — and that metabolic enzymes perform non-canonical immune regulatory functions — has fundamentally changed this view. Immunometabolism — the interdisciplinary study of bidirectional interactions between metabolic pathways and immune cell function — now stands as a distinct field integrating these previously disconnected areas of research (1, 3).

The clinical relevance of immunometabolism has grown sharply with the global rise in metabolic diseases. Obesity and type 2 diabetes involve chronic, low-grade inflammation — termed metaflammation — that contributes to insulin resistance and tissue dysfunction. Individuals with metabolic disorders face greater susceptibility to infections and worse clinical outcomes, pointing to compromised immune defense under metabolic dysregulation. Infections can, in turn, trigger or worsen metabolic abnormalities, generating vicious cycles that perpetuate both conditions (4–6). Uncovering the immunometabolic basis of these interactions may open new therapeutic avenues (7, 8).

We argue that the bidirectional nature of this crosstalk—wherein metabolic disease predisposes to infection and infection exacerbates metabolic dysfunction through shared immunometabolic pathways—represents a conceptual shift with direct therapeutic implications. Current evidence supports a model in which metabolic stress initiates low-grade inflammation through damage-associated molecular pattern (DAMP) release and inflammasome activation, which subsequently impairs insulin signaling; however, once established, insulin resistance further amplifies inflammatory signaling, creating a self-reinforcing cycle (4, 5).

This review synthesizes immunometabolism research at the intersection of metabolic and infectious diseases. We tackle several central questions: What metabolic programs govern immune cell function? How do these programs go awry in metabolic diseases? What metabolic strategies do pathogens use to evade immune defense, and how does the host fight back? Can immunometabolic pathways be targeted therapeutically to treat both classes of disease? Throughout, we emphasize controversies, flag knowledge gaps, and support all major claims with verified references.

**Figure 2:**
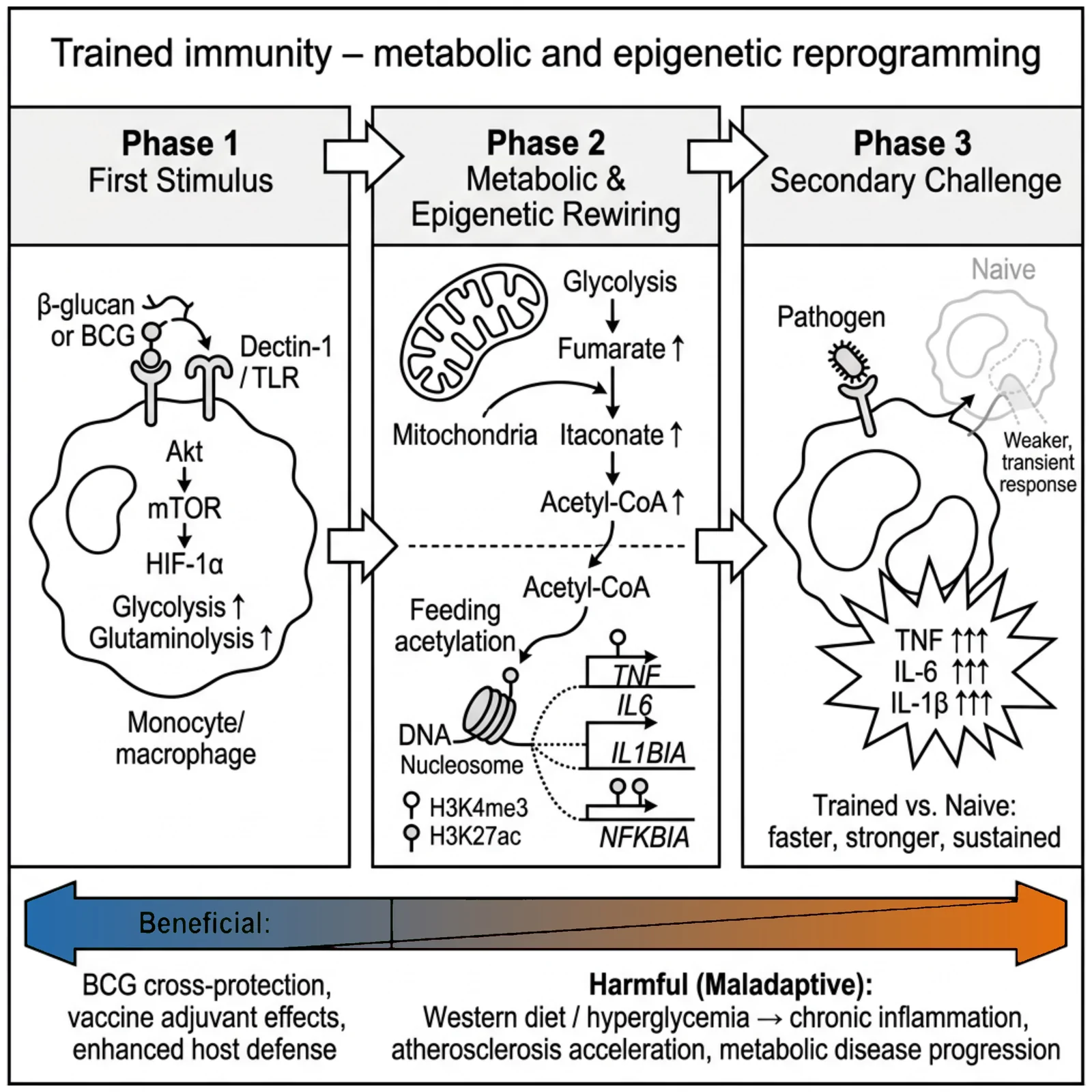
Metabolic Reprogramming in Metabolic Diseases. Multipanel overview of immunometabolic alterations linking metabolic pathology to immune dysfunction. Created with BioRender.com.

A unifying conceptual framework guides this review: The interplay between metabolic diseases and infectious diseases creates a vicious cycle that perpetuates both conditions. Metabolic dysregulation in the host creates an immunocompromised state that increases susceptibility to infections. Upon infection, the host’s metabolic responses — including glycolysis upregulation, immunometabolite production (itaconate, succinate), and epigenetic rewiring — determine infection outcomes. Unresolved infection or chronic low-grade inflammation in metabolic diseases can induce trained immunity in innate immune cells, locking them into a pro-inflammatory state that further exacerbates metabolic dysfunction. This ‘metabolic-infection-metabolic’ vicious cycle represents a key conceptual framework for understanding how immunometabolic dysfunction contributes to disease progression (4, 9, 10). The immunometabolic alterations that drive metabolic disease are illustrated in **Figure 2**.

In this review, we address the following key questions: (1) How do metabolic and infectious diseases converge on shared immunometabolic pathways? (2) What is the mechanistic basis for the bidirectional crosstalk between these disease domains? (3) How does trained immunity serve as a bridge linking metabolic and infectious disease pathogenesis? (4) What are the most promising therapeutic strategies targeting immunometabolic checkpoints, and what challenges remain for clinical translation?

## 2 Metabolic programs governing immune cell function

### 2.1 Resting versus activated immune cell metabolism

Resting immune cells primarily rely on mitochondrial oxidative phosphorylation (OXPHOS) for ATP production, a metabolic configuration appropriate for their surveillance functions, which requires sustained energy but minimal biosynthetic activity (2, 11).

Upon recognition of danger signals through pattern recognition receptors or antigen receptors, immune cells undergo rapid metabolic reprogramming characterized by a shift toward aerobic glycolysis — a phenomenon known as the Warburg effect, first described in cancer cells but now recognized as a hallmark of activated immune cells. Critically, this metabolic switch is not merely an energetic adaptation; it represents a fate-determining event. Glycolysis provides biosynthetic intermediates for nucleotide, amino acid, and lipid synthesis required for cell proliferation and effector molecule production, while also generating metabolites that directly regulate epigenetic and transcriptional programs governing immune effector functions (1, 2, 12, 13).

The molecular mechanisms governing this metabolic switch center on the PI3K/Akt/mTOR/HIF-1α axis. Phosphatidylinositol 3-kinase (PI3K) and Akt signaling promote glucose transporter translocation to the cell surface. Mechanistic target of rapamycin (mTOR) complex 1 integrates growth factor and nutrient signals to promote glycolytic enzyme expression. Hypoxia-inducible factor 1-alpha (HIF-1α), stabilized even under normoxic conditions in activated immune cells, transcriptionally activates glycolytic genes and promotes pyruvate dehydrogenase kinase expression, which inhibits pyruvate entry into the tricarboxylic acid (TCA) cycle. The metabolic configuration adopted during activation — glycolytic versus oxidative — directly determines whether an immune cell adopts a pro-inflammatory effector phenotype or a regulatory/resolving phenotype, establishing metabolic reprogramming as a cell-fate decision point rather than a mere energetic adjustment (2, 14).

### 2.2 Macrophage metabolic plasticity

Macrophages exhibit remarkable metabolic plasticity that enables them to adopt distinct functional phenotypes in response to environmental cues. The classical M1 versus M2 polarization paradigm provides a useful framework for understanding macrophage metabolic heterogeneity (15, 16).

Classically activated M1 macrophages, generated by lipopolysaccharide (LPS) and interferon-gamma (IFN-γ), undergo profound metabolic reprogramming characterized by increased glycolysis and disrupted TCA cycle function. Two critical breaks occur: at isocitrate dehydrogenase, leading to citrate accumulation for fatty acid synthesis, and at succinate dehydrogenase (SDH), resulting in succinate accumulation. Succinate stabilizes HIF-1α, promoting interleukin-1 beta (IL-1β) expression (16, 17). M1 macrophages also upregulate IRG1/ACOD1, driving itaconate production with immunomodulatory and antibacterial functions as detailed in Section 2.4 (18–20).

Alternatively activated M2 macrophages, induced by interleukin-4 (IL-4) or interleukin-13 (IL-13), maintain intact TCA cycle function and rely on OXPHOS and fatty acid oxidation (FAO) for energy production, supporting their roles in tissue repair and resolution of inflammation (15, 16). The metabolic differences between M1 and M2 macrophages are not merely correlative but causally contribute to their functional programs — pharmacological or genetic manipulation of key metabolic enzymes can skew macrophage polarization and alter inflammatory outcomes (16, 21).

### 2.3 T cell metabolic heterogeneity

T cells exhibit metabolic heterogeneity that varies by subset and differentiation state. Naive T cells are metabolically quiescent, relying primarily on OXPHOS and FAO for long-term survival. Upon antigen recognition, they differentiate into effector T cells with distinct metabolic preferences: Th1 and Th17 cells depend heavily on glycolysis and glutaminolysis, while regulatory T cells (Tregs) preferentially utilize FAO and OXPHOS, although recent studies demonstrate metabolic flexibility allowing Tregs to adapt to different tissue microenvironments (22–25).

Memory T cells exhibit enhanced FAO capacity and increased mitochondrial spare respiratory capacity (SRC), supporting their longevity and rapid recall responses. CD8+ cytotoxic T cells follow similar patterns: effector cells depend on glycolysis for cytotoxic functions, while memory cells utilize FAO for persistence. T cell metabolic programs show plasticity that allows adaptation to changing microenvironmental conditions, including nutrient availability and oxygen tension, with profound implications for anti-tumor immunity and chronic infection (22, 23, 26). The metabolic signatures of major immune cell subsets are summarized in **Table 1**.

**Table 1:** Metabolic signatures of major immune cell subsets in health and disease.

Immune cell type	Metabolic signature	Key regulatory pathways	Functional consequence
M1 macrophage	Glycolysis-dominant; truncated TCA cycle; PPP activation	HIF-1α, PKM2, mTORC1, succinate/SDH	Pro-inflammatory cytokine production (TNF-α, IL-1β, IL-6); ROS generation; bactericidal activity
M2 macrophage	OXPHOS-dominant; FAO; intact TCA cycle	PPARγ, STAT6, AMPK, IL-4/IL-13 signaling	Anti-inflammatory; tissue repair; fibrosis; arginase-1-mediated polyamine synthesis
Effector T cell (Th1/Th17)	Aerobic glycolysis; glutaminolysis; reduced FAO	MYC, HIF-1α, mTORC1, ERK/MAPK	Rapid proliferation; IFN-γ and IL-17 production; clonal expansion
Regulatory T cell (Treg)	FAO; OXPHOS; reduced glycolysis	FOXP3, AMPK, CPT1a	Immune suppression; IL-10 and TGF-β production; maintenance of self-tolerance
Memory T cell	FAO; mitochondrial respiration; spare respiratory capacity	AMPK, CPT1a, IL-15	Long-term survival; rapid recall response; stemness maintenance
Activated dendritic cell	Early glycolysis; late OXPHOS shift; FAS	TBK1-IKKϵ, HIF-1α, mTOR	Antigen presentation; co-stimulatory molecule expression (CD80/86); cytokine secretion
Neutrophil	Glycolysis-dependent; minimal mitochondrial respiration; PPP	HIF-1α, G6PD, PI3K/Akt	NETosis; ROS production (NADPH oxidase); phagocytosis; short lifespan (<24 h)
Kupffer cell (steady state)	OXPHOS-dominant; FAO; tolerogenic profile	IRF4, LXRα, PPARδ	Hepatic immune surveillance; clearance of gut-derived pathogens; tolerance maintenance
Kupffer cell (steatosis)	Glycolytic switch; succinate accumulation	HIF-1α, NLRP3, TLR4	Inflammasome activation; IL-1β release; monocyte recruitment; hepatic inflammation
Trained monocyte/macrophage	Sustained glycolysis; glutaminolysis; fumarate accumulation	Akt/mTOR/HIF-1α; H3K4me3/H3K27ac	Enhanced secondary cytokine response; epigenetic memory; cross-protection
Lipid-associated macrophage (LAM)	Lysosomal lipolysis; lipid droplet processing; low glycolysis; distinct from M1/M2	TREM2, LPL, FABP4, CD36; PPARγ/LXR axis	Lipid buffering in obese adipose tissue; potentially protective but may sustain chronic inflammation

### 2.4 Metabolites as immune regulators

Beyond their roles in energy production and biosynthesis, metabolic intermediates function as signaling molecules that directly regulate immune cell function (16, 27, 28).

Recent work identified histone lactylation as a new epigenetic mechanism by which lactate directly regulates macrophage gene expression (29).

Succinate, accumulating in LPS-activated macrophages due to TCA cycle disruption, inhibits prolyl hydroxylase enzymes, preventing HIF-1α degradation and promoting inflammatory gene expression. Extracellularly, succinate activates its receptor SUCNR1 (GPR91), promoting dendritic cell migration and enhancing inflammatory responses (17, 27, 30).

Itaconate, produced by IRG1/ACOD1 in activated macrophages, was initially recognized for antibacterial properties but is now appreciated as an important immunomodulatory metabolite. Itaconate activates Nrf2 by alkylating KEAP1, leading to antioxidant and anti-inflammatory gene expression, and inhibits SDH, creating a negative feedback loop that limits succinate accumulation and inflammatory signaling (18–20).

The mitochondrial enzyme aconitate decarboxylase 1 (ACOD1, also known as immunoresponsive gene 1, IRG1) catalyzes itaconate synthesis from cis-aconitate and is among the most highly upregulated genes in activated macrophages. ACOD1/IRG1 expression is driven by type I and type II interferon signaling, TLR ligands, and inflammatory stimuli, positioning it as a central immunometabolic checkpoint. Beyond its canonical antibacterial function—mediated by itaconate’s inhibition of bacterial isocitrate lyase in the glyoxylate shunt—ACOD1/IRG1-derived itaconate exerts broad anti-inflammatory effects through multiple mechanisms: alkylation of KEAP1 to activate NRF2-dependent antioxidant responses, inhibition of succinate dehydrogenase (SDH) to suppress mitochondrial reverse electron transport and reactive oxygen species production, and attenuation of NLRP3 inflammasome activation. ACOD1 plays a dual role in infection: while protective in bacterial sepsis and viral infections by limiting excessive inflammation, sustained ACOD1 activation can impair bacterial clearance by suppressing glycolysis-dependent macrophage bactericidal activity, exemplifying the context-dependent nature of immunometabolic regulation.

Fatty acids and their derivatives serve as important immune regulators. Saturated fatty acids promote inflammatory responses through Toll-like receptor 4 (TLR4) activation, while omega-3 fatty acids exert anti-inflammatory effects through PPARγ and GPR120 (FFA4) (31, 32).

Amino acid metabolism also shapes immune function. Arginine metabolism represents a key bifurcation: M1 macrophages express inducible nitric oxide synthase (iNOS), converting arginine to nitric oxide, whereas M2 macrophages express arginase 1, promoting tissue repair. Tryptophan metabolism through indoleamine 2,3-dioxygenase (IDO) produces kynurenine, suppressing effector T cell function and promoting Treg differentiation partly through aryl hydrocarbon receptor (AhR) activation (33, 34).

## 3 Trained immunity and metabolic memory

### 3.1 Mechanisms of trained immunity

Trained immunity describes the phenomenon whereby innate immune cells — the body’s first line of defense — exhibit long-lasting functional reprogramming following exposure to specific stimuli, enabling enhanced responses to subsequent challenges. This concept challenges the traditional view that immunological memory is exclusive to adaptive immunity (35–37).

The molecular mechanisms involve coordinated metabolic and epigenetic reprogramming. β-glucan, a fungal cell wall component, is a well-characterized inducer acting through Dectin-1. β-glucan stimulation triggers sustained metabolic reprogramming in monocytes and macrophages, characterized by increased glycolysis, glutaminolysis, and TCA cycle remodeling. These metabolic changes are intimately linked to epigenetic modifications that maintain the trained state. Accumulation of TCA cycle intermediates — fumarate and acetyl-CoA — influences epigenetic enzyme activity. Fumarate inhibits lysine demethylases, increasing histone methylation at inflammatory gene promoters. Acetyl-CoA serves as a substrate for histone acetyltransferases, promoting gene activation. The result is a chromatin configuration enabling enhanced transcriptional responses upon restimulation (14, 38–40).

An important unresolved question concerns the duration of trained immunity. BCG vaccination provides nonspecific protection against unrelated pathogens for approximately one year; however, the duration induced by other stimuli such as βglucan or oxidized LDL remains unclear. A 2025 review in Cell Research has further elaborated the role of inflammatory memory in disease pathogenesis, providing additional conceptual frameworks that align with the trained immunity model (41). Longitudinal studies with repeated metabolic and epigenetic profiling are needed to define the kinetics of trained immunity and to identify mechanisms that maintain versus terminate the trained state (42, 43).

**Figure 3:**
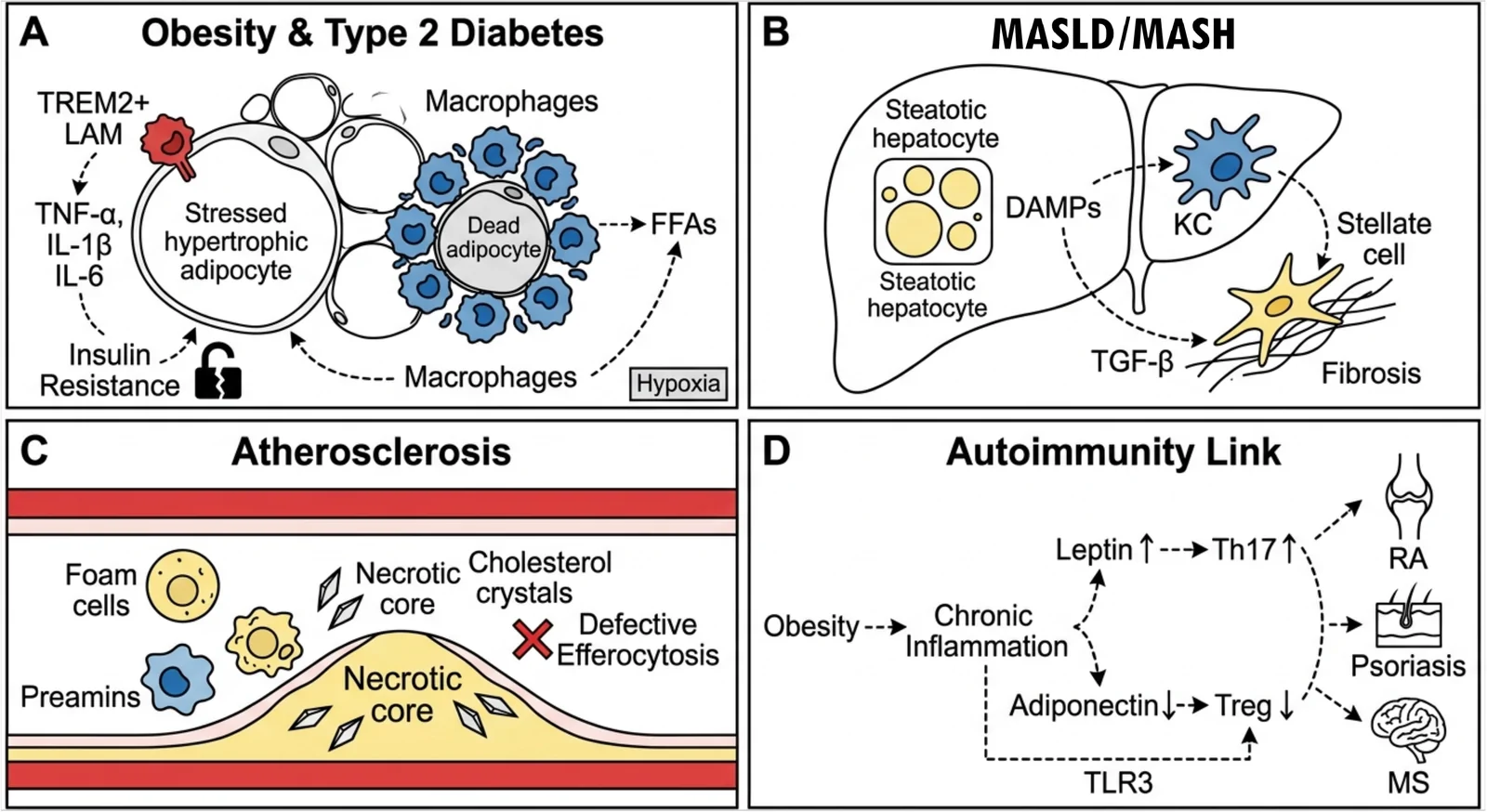
**(A–D)** Trained immunity: metabolic and epigenetic reprogramming. Three-phase flow diagram illustrating trained immunity in monocytes/macrophages. Phase 1 (First Stimulus): beta-glucan or BCG vaccine engages Dectin-1/TLR receptors, activating Akt-> mTOR-> HIF-1α signaling, driving upregulated glycolysis and glutaminolysis. Phase 2 (Metabolic and Epigenetic Rewiring): mitochondrial accumulation of fumarate and itaconate, increased acetyl-CoA feeding histone acetylation (H3K4me3, H3K27ac) at promoters of pro-inflammatory genes (TNF, IL-6, IL1BIA, NFKBIA). Phase 3 (Secondary Challenge): upon pathogen encounter, trained cells mount faster, stronger, and sustained cytokine responses compared to naive cells. Bottom continuum indicates spectrum from beneficial (BCG cross-protection, vaccine adjuvant effects, enhanced host defense) to harmful/maladaptive outcomes (Western diet/hyperglycemia-> chronic inflammation, atherosclerosis acceleration). Created with BioRender.com.

The metabolic and epigenetic reprogramming underlying trained immunity is illustrated in **Figure 3**.

### 3.2 Maladaptive trained immunity in metabolic disease

While trained immunity can provide protective benefits, maladaptive trained immunity describes situations where persistent metabolic or inflammatory stimuli induce long-lasting pro-inflammatory reprogramming that contributes to chronic disease (35, 44).

In metabolic diseases, persistent exposure to metabolic danger signals — oxidized LDL in atherosclerosis or high glucose in diabetes — may induce trained immunity in monocytes and macrophages, locking them into a pre-activated, pro-inflammatory state that perpetuates chronic inflammation and tissue damage. This may explain why metabolic diseases feature sustained innate immune hyperreactivity even after the initial metabolic insult has been addressed (39, 45). The therapeutic challenge lies in enhancing beneficial training while preventing or reversing maladaptive training, representing an important frontier in immunometabolic therapy. However, this goal is complicated by the lack of reliable biomarkers that distinguish protective from pathological trained immunity in clinical settings, and by the fact that most evidence for maladaptive training derives from *in vitro* or ex vivo stimulation assays that may not fully recapitulate *in vivo* conditions.

The concept of trained immunity provides a mechanistic bridge between metabolic disorders and infection susceptibility. As detailed in the following sections, metabolic diseases create a permissive environment for maladaptive trained immunity, while infections can initiate or exacerbate metabolic dysfunction through trained immunity-mediated inflammatory amplification.

## 4 Immunometabolism in metabolic diseases

### 4.1 Obesity and type 2 diabetes: adipose tissue as an immunometabolic hub

Obesity is characterized by chronic, low-grade inflammation in adipose tissue that contributes to systemic insulin resistance. In lean individuals, adipose tissue contains predominantly anti-inflammatory immune cell populations — M2-like macrophages, regulatory T cells, and type 2 innate lymphoid cells — that maintain homeostasis. During obesity, adipocyte hypertrophy, hypoxia, endoplasmic reticulum stress, and cell death trigger recruitment of circulating monocytes and their differentiation into pro-inflammatory macrophages. M1-like macrophages accumulate, forming crown-like structures around dead adipocytes and producing TNF-α, IL-1β, and IL-6, which interfere with insulin signaling (4–6, 15, 31, 46).

A critical question remains: whether inflammation causes insulin resistance or represents a secondary response to metabolic overload. The causal hypothesis gained support from the CANTOS trial, which demonstrated that IL-1β blockade with canakinumab modestly improved glycemic parameters, and from genetic studies showing that deletion of inflammatory signaling components protects against diet-induced insulin resistance in mice. However, Mendelian randomization studies have yielded mixed results: a recent large-scale analysis found that genetically predicted circulating IL-6 levels have a modest causal effect on insulin resistance, yet the effect size was substantially smaller than that of adiposity itself, suggesting that inflammation acts more as an amplifier than a primary driver. Insulin resistance can also develop in the absence of overt inflammation in some models, and anti-inflammatory interventions produce only partial metabolic improvement (6, 47, 48).

The emerging consensus positions inflammation as an amplifier rather than the sole cause of insulin resistance, though disentangling causation from correlation in human studies remains methodologically challenging, as most evidence derives from cross-sectional or short-term intervention designs rather than longitudinal metabolic-immune co-profiling. Metabolic overload — driven by excessive nutrient intake — initiates insulin resistance through lipid intermediate accumulation, mitochondrial dysfunction, and endoplasmic reticulum stress. Inflammation then amplifies and sustains these defects, creating a self-reinforcing loop. Single-cell sequencing has identified novel macrophage subsets in obese adipose tissue, including lipid-associated macrophages (LAMs) expressing high TREM2 levels; whether LAMs are protective or pathogenic remains under active investigation (5, 6, 49). Distinct from classical M1 or M2 macrophages, LAMs exhibit a unique metabolic signature characterized by enhanced lysosomal lipolysis, active lipid droplet processing, and a transcriptional program dominated by lipid handling genes (Lpl, Fabp4, Cd36) rather than canonical inflammatory or repair markers. This lipid-scavenging metabolic profile allows LAMs to buffer toxic lipid species in obese adipose tissue, although their chronic activation may paradoxically sustain inflammatory signaling through TREM2-dependent pathways.

Adipose tissue also serves as an endocrine organ, releasing adipokines that shape systemic immune balance. Leptin, elevated in obesity, promotes Th1 and Th17 differentiation while suppressing Treg function through mTOR pathway activation, tilting the immune system toward pro-inflammatory phenotypes. Adiponectin, depressed in obesity, normally exerts anti-inflammatory effects; its deficiency may increase autoimmune susceptibility. Resistin drives macrophage inflammation through TLR4 signaling, while visfatin activates NF-κB and stimulates matrix metalloproteinase production — mechanisms directly implicated in rheumatoid arthritis pathogenesis. The resulting Th17/Treg imbalance represents a key link between obesity and autoimmune disease predisposition (50–52).

### 4.2 Tissue-specific immunometabolic dysregulation

In the liver, MASLD progresses to MASH through immunometabolic mechanisms. Hepatocyte lipid accumulation causes mitochondrial dysfunction, reactive oxygen species production, and release of damage-associated molecular patterns (DAMPs). These signals activate Kupffer cells, which recruit monocyte-derived macrophages and activate hepatic stellate cells, promoting hepatocyte injury and fibrosis. The NLRP3 inflammasome serves as a central sensor linking metabolic stress to hepatic inflammation (53, 54).

In skeletal muscle, intramyocellular lipid accumulation activates protein kinase C theta, impairing insulin signaling. Macrophage infiltration correlates with insulin resistance, and muscle-derived cytokines (myokines) influence systemic metabolism through endocrine mechanisms (55).

In pancreatic islets, amyloid deposition, oxidative stress, and lipotoxicity trigger local inflammation that impairs β-cell function and promotes β-cell death — termed islet metaflammation — contributing to the progressive decline in insulin secretion characteristic of type 2 diabetes (6).

### 4.3 Atherosclerosis as an immunometabolic disease

Atherosclerosis is now recognized as a chronic inflammatory disease driven by metabolic abnormalities. Retention and modification of lipoproteins within the arterial wall initiates an inflammatory response that, when unresolved, leads to plaque formation. Macrophages play central roles: monocytes recruited to the arterial intima differentiate into macrophages that take up modified lipoproteins, becoming foam cells. Within the hypoxic, acidic, and nutrient-limited plaque microenvironment, macrophages undergo metabolic reprogramming toward glycolysis (56–58).

A critical defect in atherosclerotic macrophages is impaired efferocytosis — the clearance of dead cells — leading to secondary necrosis and expansion of the necrotic core, a hallmark of rupture-prone plaques. Cholesterol crystals activate the NLRP3 inflammasome, promoting IL-1β and IL-18 production. The landmark CANTOS trial established proof-of-concept that targeting inflammation — specifically IL-1β blockade with canakinumab — reduces cardiovascular events independent of lipid-lowering (47, 48, 54, 56, 57).

Building on CANTOS, more recent trials have expanded the therapeutic landscape. The COLCOT and LoDoCo2 trials demonstrated that low-dose colchicine — which broadly inhibits inflammasome activation, microtubule polymerization, and neutrophil function — significantly reduces major adverse cardiovascular events in patients with recent myocardial infarction or chronic coronary disease. These results led to FDA approval of colchicine for cardiovascular prevention in 2023, though subsequent analyses have debated the magnitude of benefit in broader populations and raised questions about generalizability to non-Western cohorts. Beyond NLRP3, targeting gasdermin D-mediated pyroptosis is a promising strategy: preclinical studies using disulfiram, a gasdermin D pore formation inhibitor, have shown reduced necrotic core expansion and improved plaque stability. The NLRP3-specific inhibitor MCC950 and its derivatives have demonstrated efficacy in preclinical atherosclerosis models, and several oral NLRP3 inhibitors (e.g., DFV890, ZYIL1) are currently in phase 2 clinical trials for cardiovascular indications (59–64).

Additionally, the CANTOS sub-study revealed that IL-1β blockade preferentially benefited patients with high baseline clonal hematopoiesis of indeterminate potential (CHIP), particularly TET2 mutations, linking somatic mutations in hematopoietic cells to inflammasome-driven atherosclerosis — a finding that opens new avenues for precision immunometabolic cardiovascular therapy (65, 66).

**Figure 4:**
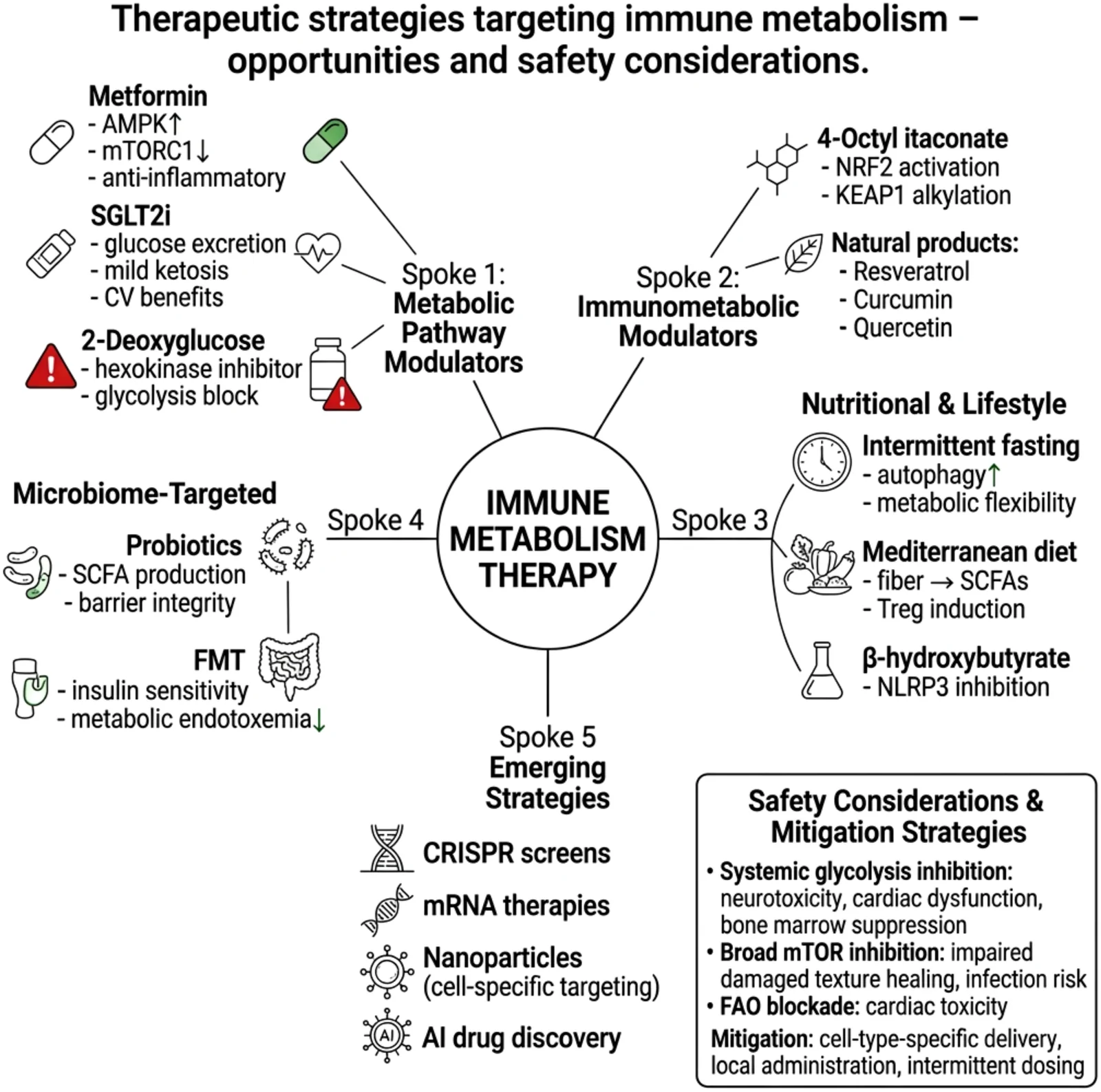
Host-pathogen metabolic competition in infection. Schematic depiction of the metabolic battlefield at the host-pathogen interface. Top panel shows host defense mechanisms: macrophage glycolysis-> ROS production; neutrophil glycolysis-> extracellular trap (NET) formation; T cell glutaminolysis-> cytokine responses. The central arena features competition for itaconate (antibacterial molecule), succinate, and HIF-1α-regulated pathways. Bottom panels illustrate pathogen-specific strategies: Bacteria (Mycobacterium tuberculosis blocks phagosome acidification; Staphylococcus aureus triggers acute glycolysis then promotes FAO-mediated immunosuppression; Klebsiella pneumoniae employs Type VI secretion); Viruses (HIV depletes CD4+ T cells via glucose hijacking; SARS-CoV-2 exploits metabolic comorbidities); Fungi/Parasites (Candida albicans uses metabolic flexibility; Leishmania drives M2-like phenotype). Created with BioRender.com.

The immunometabolic dysregulation described above extends beyond chronic inflammation to directly compromise host defense. Hyperglycemia blunts neutrophil chemotaxis and phagocytosis; adipose tissue inflammation diverts immune resources from infection surveillance; insulin resistance alters T cell metabolic programming, reducing their capacity to mount effective responses. Chronic low-grade inflammation also primes innate immune cells toward a hyperresponsive yet functionally exhausted state, paradoxically increasing susceptibility to severe infections while sustaining tissue-damaging inflammation. These converging pathways create an immunocompromised milieu that pathogens readily exploit. The key metabolic alterations in obesity, MASLD/MASH, and atherosclerosis are summarized in **Figure 4** (67).

## 5 Immunometabolism in infectious diseases

Infection represents a fundamental competition between host and pathogen for metabolic resources. Pathogens must acquire nutrients from the host to support replication, while the host employs metabolic strategies to restrict pathogen access to essential nutrients — a concept termed nutritional immunity (9).

Iron sequestration is a classic example: the host produces transferrin and lactoferrin to reduce free iron availability, starving pathogens of this essential cofactor. In response, pathogens have evolved sophisticated iron acquisition systems, including siderophores and transport systems that extract iron from host proteins. Beyond nutrient restriction, the host actively reprograms immune cell metabolism: macrophage glycolysis supports production of reactive oxygen species and nitric oxide, while itaconate directly inhibits bacterial isocitrate lyase (68, 69).

### 5.1 Bacterial infections

Mycobacterium tuberculosis (Mtb) engages in complex metabolic interplay with infected macrophages. To control Mtb, macrophages polarize toward an M1 phenotype with glycolysis, nitric oxide production, and itaconate accumulation. Itaconate inhibits Mtb isocitrate lyase, required for bacterial persistence under hypoxic conditions within granulomas. However, Mtb counters by utilizing host cholesterol and fatty acids through the Mce4 transporter system and by manipulating host macrophage metabolism to inhibit phagolysosome acidification (18).

The metabolic strategies employed by bacterial pathogens reveal remarkable diversity. Mycobacterium tuberculosis preferentially utilizes host-derived fatty acids and cholesterol as carbon sources within the phagosome, while simultaneously inhibiting phagosome-lysosome fusion to evade degradation; this metabolic adaptation is regulated by the ICL1 and ICL2 isocitrate lyase isoforms that enable growth on C2 substrates. Salmonella enterica serovar Typhimurium exploits the host inflammatory response by utilizing tetrathionate as a terminal electron acceptor, gaining a growth advantage over fermenting commensals in the inflamed gut, and scavenges host-derived itaconate to promote intracellular replication within macrophages. Staphylococcus aureus employs a biphasic metabolic strategy: during acute infection, it relies on glycolysis for rapid proliferation and toxin production, but during biofilm formation and chronic infection, it shifts to fatty acid β-oxidation and the tricarboxylic acid cycle, conferring antibiotic tolerance. Understanding these pathogen-specific metabolic strategies provides a foundation for developing metabolism-targeted antimicrobial interventions.

Staphylococcus aureus exhibits metabolic flexibility contributing to its success as both acute and chronic pathogen. During acute infection, S. aureus triggers robust glycolytic responses required for neutrophil extracellular trap formation and bactericidal activity. During chronic or biofilm-associated infections, S. aureus can promote a more oxidative metabolic state in host cells, creating an immunosuppressive environment conducive to bacterial persistence (70).

Klebsiella pneumoniae, particularly hypervirulent strains, causes severe pneumonia and sepsis by inducing profound metabolic stress in the lung, using type VI secretion systems to compete for nutrients. The resulting metabolic disruption impairs immune cell function and contributes to high mortality. This bidirectional dynamic — pathogen exploitation of metabolic substrates and infection-exacerbated metabolic dysfunction — illustrates the interplay discussed in Section 1: bacterial pathogens exploit host nutrient stores abnormally abundant in metabolic disease, while the ensuing inflammatory response exacerbates insulin resistance and metabolic dysfunction.

### 5.2 Viral infections

Viruses, lacking independent metabolism, must extensively reprogram host cell metabolism to support replication — creating vulnerabilities that can be therapeutically exploited (9).

Human immunodeficiency virus (HIV) causes profound immune dysfunction despite effective antiretroviral therapy. HIV depletes CD4+ T cells and causes metabolic exhaustion in surviving T cells, characterized by mitochondrial dysfunction, increased oxidative stress, and reduced glycolytic capacity. Monocytes and macrophages serving as viral reservoirs remain metabolically active, contributing to chronic inflammation that accelerates aging and increases cardiovascular complications (71, 72).

The COVID-19 pandemic dramatically illustrated the impact of metabolic health on infection outcomes. Obesity and diabetes emerged as among the strongest risk factors for severe COVID-19, with these individuals displaying elevated glycolysis in monocytes, mitochondrial stress, and low-grade inflammation at baseline. Upon SARS-CoV-2 infection, these features are amplified, contributing to cytokine storm and multiorgan failure (7, 8).

Of particular concern is Long COVID (post-acute sequelae of SARS-CoV-2 infection), which affects a substantial proportion of survivors. Emerging evidence demonstrates that Long COVID involves persistent immunometabolic dysregulation and mitochondrial dysfunction. Multi-omics studies have identified sustained mitochondrial fragmentation, impaired fatty acid oxidation, and reduced mitochondrial respiratory capacity in peripheral blood mononuclear cells from Long COVID patients months after viral clearance. Metabolomic profiling reveals depleted tryptophan and serotonin levels, elevated circulating succinate, and persistent activation of the kynurenine pathway — a metabolic signature associated with chronic fatigue and neurocognitive symptoms. SARS-CoV-2 can directly compromise mitochondrial function by downregulating mitochondrial gene expression and inducing mitophagy. The resulting bioenergetic crisis creates a state of metabolic exhaustion in immune cells, impairing their capacity to resolve inflammation and predisposing patients to secondary complications. These metabolic abnormalities correlate with persistent exercise intolerance and post-exertional malaise, and preliminary evidence from small open-label studies suggests that interventions targeting mitochondrial biogenesis, such as NAD+ precursor supplementation, may ameliorate some Long COVID symptoms, though this requires validation in randomized trials. These findings collectively demonstrate how a viral infection can leave lasting metabolic scars — a phenomenon we term ‘post-infectious metabolic sequelae’ — that may perpetuate chronic metabolic dysfunction (73–76).

Epstein-Barr virus (EBV) reprograms host methionine metabolism, affecting S-adenosylmethionine levels and thereby influencing histone and DNA methylation patterns that maintain viral latency. Influenza viruses rely heavily on host glycolysis and fatty acid synthesis for replication; inhibition of these pathways reduces viral replication in preclinical models, suggesting potential for host-directed antiviral therapies.

SARS-CoV-2 infection is a particularly instructive model of immunometabolic dysregulation. The virus exploits host glycolytic machinery for replication while simultaneously impairing mitochondrial function and inducing macrophage metabolic reprogramming toward a hyperinflammatory state. Pre-existing metabolic comorbidities — obesity, type 2 diabetes, and hypertension — rank among the strongest risk factors for severe COVID-19, consistent with the bidirectional framework outlined in Section 1. Preliminary longitudinal studies — many limited by small sample sizes and lack of appropriate controls — have reported persistent metabolic abnormalities in Long COVID patients, including sustained insulin resistance, dyslipidemia, mitochondrial dysfunction, and altered serotonin metabolism, that can endure for over a year post-infection. These findings show how acute infections can leave lasting immunometabolic scars that perpetuate chronic disease.

### 5.3 Fungal and parasitic infections

Candida albicans exhibits remarkable metabolic flexibility, enabling it to survive in diverse host niches. Neutrophils and macrophages upregulate glycolysis to produce reactive oxygen species and antifungal peptides; C. albicans counters by utilizing alternative carbon sources, including fatty acids and amino acids, to survive within the hypoxic environment of phagocytes (77).

Leishmania parasites actively manipulate host macrophage metabolism, driving macrophages toward an M2-like phenotype characterized by arginase expression and polyamine production that support parasite replication, while also modulating host lipid metabolism to acquire essential lipids (78).

### 5.4 Micronutrients in immune metabolism

Iron is essential for T cell proliferation, serving as a cofactor for ribonucleotide reductase. During infection, the host produces hepcidin to reduce iron availability to pathogens, but excessive restriction can impair immune cell function. Zinc serves as a cofactor for over 300 enzymes, enhances interferon signaling and antiviral immunity, and zinc deficiency increases infection susceptibility. During infection, the host sequesters zinc through metallothionein induction, limiting pathogen access (68, 69, 79, 80).

Vitamin D promotes a tolerogenic macrophage phenotype, enhances autophagy for intracellular pathogen clearance, and regulates expression of antimicrobial peptides including cathelicidin (81, 82). Vitamin A and retinoic acid are critical for T cell homing to the gut and the balance between regulatory and effector T cell differentiation (83).

### 5.5 Infection-induced trained immunity and post-infectious metabolic sequelae

Infections do not merely exploit pre-existing metabolic vulnerabilities; they can induce lasting immunometabolic changes that exacerbate or even initiate metabolic disease. Acute infections trigger systemic metabolic reprogramming — including hepatic insulin resistance, adipose tissue lipolysis, and skeletal muscle catabolism — as part of the host defense response. While adaptive in the short term, repeated or severe infections can leave lasting marks on the immune system through trained immunity and epigenetic remodeling of hematopoietic stem cells (9, 10).

The concept of ‘post-infectious metabolic sequelae’ describes the phenomenon whereby infection-induced trained immunity and metabolic reprogramming persist long after pathogen clearance, creating a lasting metabolic vulnerability. In the context of severe COVID-19, longitudinal studies have demonstrated that metabolic abnormalities — including insulin resistance, dyslipidemia, and mitochondrial dysfunction — can persist for over a year in some individuals. This is not unique to SARS-CoV-2: sepsis survivors show increased long-term risk of type 2 diabetes and cardiovascular events, an effect partly mediated by persistent epigenetic changes at inflammatory gene loci in bone marrow progenitors. Similarly, chronic viral infections such as HIV accelerate metabolic aging through sustained innate immune activation and trained immunity (10, 73, 74, 84).

The gut microbiome serves as an additional interface in this pathological loop. Infection-induced dysbiosis alters short-chain fatty acid (SCFA) production, compromising intestinal barrier integrity and promoting metabolic endotoxemia. Reduced butyrate-producing bacteria impair histone deacetylase inhibition in intestinal immune cells, weakening the gut barrier and allowing translocation of bacterial lipopolysaccharide into the systemic circulation. This metabolic endotoxemia, in turn, activates TLR4 signaling in adipose tissue macrophages and hepatocytes, perpetuating insulin resistance and hepatic steatosis (85–89).

Critically, the metabolic-infection vicious cycle operates bidirectionally. Metabolic diseases create an immunocompromised milieu that increases infection susceptibility, while infections — through trained immunity, epigenetic reprogramming, mitochondrial damage, and gut dysbiosis — leave lasting metabolic sequelae that accelerate or initiate metabolic dysfunction. This circular relationship demands integrated therapeutic strategies targeting both metabolic and infectious disease processes concurrently. The host-pathogen metabolic competition across different pathogen classes is illustrated in **Figure 3**.

## 6 Therapeutic strategies targeting immune metabolism

### 6.1 Repurposed metabolic drugs

An overview of therapeutic strategies targeting immune metabolism is provided in **Table 2**. Metformin, the first-line medication for type 2 diabetes, activates AMPK, inhibiting mTORC1 and promoting mitochondrial function. These effects reduce M1 macrophage polarization, enhance Treg function, and exert anti-inflammatory actions. Epidemiological studies suggest metformin use is associated with reduced risk of infectious diseases and improved cancer outcomes, though these observational findings are subject to confounding by indication and require validation in randomized trials (90).

**Table 2:** Therapeutic strategies targeting immune metabolism.

Therapeutic strategy	Molecular target	Mechanism of action	Key clinical evidence
Metformin	Mitochondrial Complex I; AMPK	Inhibits mitochondrial respiration; activates AMPK; reduces hepatic gluconeogenesis; AMPK-mediated anti-inflammatory signaling	UKPDS (T2DM cardiovascular benefit); TAME trial (aging); COVID-19 observational studies
SGLT2 inhibitors (empagliflozin, dapagliflozin)	SGLT2 (renal)	Glycosuria-induced ketogenesis → β-hydroxybutyrate elevation; NLRP3 inflammasome inhibition; AMPK/SIRT1/PGC-1α axis activation	EMPA-REG OUTCOME (CV death reduction); DAPA-HF; DELIVER (HFpEF benefit); DAPA-CKD (renal protection)
mTOR inhibitors (rapamycin/everolimus)	mTORC1	Inhibits mTORC1-driven glycolytic shift; promotes autophagy and mitochondrial biogenesis; enhances Treg differentiation; reduces senescence-associated secretory phenotype	Transplant immunosuppression; anti-aging models; autoimmune disease trials; lymphoma (mTOR-driven)
Colchicine	Tubulin polymerization; NLRP3 inflammasome	Blocks microtubule polymerization → inhibits NLRP3 inflammasome assembly and IL-1β processing; reduces neutrophil chemotaxis	COLCOT (post-MI CV benefit); LoDoCo2 (chronic CAD); CANTOS (canakinumab IL-1β proof-of-concept)
GLP-1 receptor agonists (semaglutide, liraglutide)	GLP-1R	Weight loss; anti-inflammatory cytokine shift; improved insulin sensitivity; direct immune cell GLP-1R signaling; reduced hepatic steatosis	SELECT (semaglutide, CV benefit in obesity); LEADER (liraglutide); STEP trials (weight loss)
Itaconate derivatives (4-OI, DMI, itaconate)	KEAP1/NRF2; SDH; NLRP3; GAPDH	Electrophilic alkylation of KEAP1 → NRF2 activation; SDH inhibition → reduced succinate-driven IL-1β; GAPDH inhibition → glycolytic blockade	Preclinical: sepsis models; ischemia-reperfusion injury; psoriasis models; MS models
Apabetalone (RVX-208)	BET proteins (BRD2/BRD3/BRD4)	Epigenetic modulation: inhibits BET bromodomain-mediated transcription of inflammatory genes (NF-κB-dependent); raises ApoA-I/HDL	BETonMACE (phase III, modest MACE reduction in T2DM + ACS subgroup); SUSTAIN/ASSURE (HDL effects)
Canakinumab (anti-IL-1β mAb)	IL-1β	Neutralizes IL-1β, disrupting NLRP3 → IL-1β → IL-6 → CRP inflammatory cascade; no effect on lipid levels	CANTOS (atherosclerosis, CV event reduction independent of lipid lowering; increased fatal infection risk)
Fasting regimens/CR/IF	AMPK; SIRT1; mTOR; autophagy	Nutrient deprivation → AMPK activation; mTOR suppression; SIRT1-mediated deacetylation; enhanced autophagy and mitophagy; reduced oxidative stress	CALERIE (CR safety); observational/interventional: improved insulin sensitivity; reduced inflammatory markers; bone marrow immune memory effects
Probiotics/FMT	Gut microbiota composition and metabolites	SCFA (butyrate, propionate) production → HDAC inhibition; Treg induction; intestinal barrier integrity; bile acid metabolism modulation	Recurrent C. difficile (FMT efficacy >90%); ulcerative colitis trials; metabolic syndrome RCTs; emerging oncology applications
CRISPR-based immunometabolic editing	Cell-specific metabolic genes (e.g., PKM2, HIF1α)	Gene disruption or activation of metabolic checkpoints in immune cells; CAR-T metabolic engineering; *in vivo* delivery (LNP, AAV)	Preclinical; CAR-T Phase I/II (metabolic-enhanced persistence); LNP-mRNA Phase III (vaccines); sickle cell (CTX001, approved)

sodium-glucose cotransporter-2 (SGLT2) inhibitors, which lower blood glucose by promoting urinary glucose excretion, have emerged as particularly promising immunometabolic modulators. Beyond glycemic control, these agents induce mild ketosis, shift systemic fuel utilization toward fatty acid oxidation and ketone body production, and directly suppress NLRP3 inflammasome activation in renal and cardiac macrophages through mechanisms involving AMPK activation and reduced mTORC1 signaling. The cardiovascular and renal benefits demonstrated in landmark trials (EMPA-REG OUTCOME, DAPA-HF, DELIVER) extend substantially beyond glucose lowering. Recent studies reveal that canagliflozin impairs T cell effector function through metabolic suppression, suggesting potential applications in T cell-mediated autoimmune diseases. Canagliflozin also reduces IL-17 production in CD4+ T cells, linking SGLT2 inhibition to modulation of the Th17/Treg axis — a mechanism with implications for both metabolic and autoimmune diseases (91–94).

Clinical trials have begun to validate immunometabolic targeting in cardiovascular disease. The CANTOS trial demonstrated that canakinumab, an IL-1β neutralizing antibody, reduced cardiovascular events by 15% in patients with prior myocardial infarction, providing proof-of-concept that anti-inflammatory therapy targeting innate immune pathways can improve clinical outcomes independently of lipid-lowering. The COLCOT and LoDoCo2 trials further showed that colchicine, a microtubule inhibitor with broad anti-inflammatory effects, reduced cardiovascular events by 23-31% in patients with coronary artery disease, with its mechanism partially attributed to inhibition of NLRP3 inflammasome activation and neutrophil immunometabolic reprogramming. SGLT2 inhibitors have demonstrated cardiorenal benefits beyond glucose lowering: EMPA-REG OUTCOME, DAPA-HF, and DELIVER trials collectively showed that empagliflozin and dapagliflozin reduced heart failure hospitalizations and cardiovascular death, with emerging evidence suggesting these benefits are mediated partly through immunometabolic mechanisms including ketone body signaling, reduced macrophage inflammation, and attenuated NLRP3 inflammasome activation.

Glycolysis inhibitors such as 2-deoxyglucose (2-DG) have been investigated for suppressing overactivated immune cells. While short-term 2-DG treatment can selectively inhibit effector T cells and M1 macrophages, its clinical translation has largely stalled due to dose-limiting toxicities, including cardiotoxicity and bone marrow suppression observed at therapeutic doses. Consequently, research interest has shifted toward more selective glycolytic inhibitors and cell-type-specific delivery strategies (95).

### 6.2 Novel immunometabolic modulators

Itaconate derivatives represent a leading class of novel immunometabolic therapeutics. Native itaconate has poor cellular permeability, but cell-permeable derivatives — 4-octylitaconate (4-OI), dimethyl itaconate (DMI), and itaconate-alkyne probes — have demonstrated potent anti-inflammatory activity across diverse preclinical models. 4-OI exerts protective effects through multiple mechanisms: Nrf2 activation via KEAP1 alkylation, succinate dehydrogenase inhibition, NLRP3 inflammasome suppression, and inhibition of the glycolytic enzyme GAPDH. In preclinical models of sepsis, acute lung injury, autoimmune encephalitis, and viral pneumonia, 4-OI reduces inflammatory cytokine production and mitigates tissue damage. Recent studies have shown that 4-OI can also modulate T cell differentiation, promoting Treg function while restraining Th17 responses — extending itaconate’s therapeutic potential beyond innate immunity. Several pharmaceutical companies have initiated development programs for itaconate-based therapeutics, with modified prodrugs and sustained-release formulations under investigation. A key challenge for clinical translation remains the balance between the anti-inflammatory and potential immunosuppressive effects of sustained itaconate pathway activation. Additionally, most preclinical data derive from rodent models or cell culture systems, and the pharmacokinetic properties of current derivatives — including rapid hydrolysis and off-target electrophilic reactions — may limit therapeutic applicability. No itaconate derivative has yet entered clinical trials, underscoring the gap between preclinical promise and clinical reality (18, 96–99).

Natural products continue to be explored for immunometabolic modulation. Resveratrol activates SIRT1 with metabolic and anti-inflammatory benefits; curcumin inhibits NF-κB signaling; quercetin and other flavonoids modulate immune cell metabolism through multiple mechanisms. While preclinical data are encouraging, clinical utility is limited by bioavailability challenges, spurring development of nanoparticle-encapsulated formulations and synthetic analogs (100).

### 6.3 Nutritional and lifestyle interventions

Intermittent fasting and calorie restriction enhance metabolic flexibility, increase autophagy, reduce baseline inflammatory markers, and improve insulin sensitivity. β-hydroxybutyrate, a ketone body produced during fasting, directly blocks NLRP3 inflammasome activation, providing a mechanistic link between dietary interventions and reduced inflammation (101–103).

The Mediterranean diet, rich in fiber, polyphenols, and unsaturated fats, exerts anti-inflammatory effects through immunometabolic regulation. Gut bacteria ferment dietary fiber into short-chain fatty acids (SCFAs) — acetate, propionate, and butyrate — which function as histone deacetylase inhibitors and ligands for GPR41/GPR43, promoting intestinal Treg differentiation and reducing systemic inflammation (87–89, 104).

### 6.4 Microbiome-targeted therapies

The gut microbiome serves as a major extrinsic regulator of host immune metabolism. Microbial dysbiosis is closely linked to metabolic endotoxemia and insulin resistance. Supplementation with specific probiotics or their fermentation products can improve intestinal barrier function and modulate local and systemic immune responses. Fecal microbiota transplantation (FMT) has been shown to transiently improve insulin sensitivity in patients with metabolic syndrome, an effect driven by baseline microbiota composition of the recipient (85, 105, 106).

### 6.5 Safety considerations and emerging cell-type-specific strategies

Targeting core metabolic pathways carries inherent safety risks, as glycolysis, the TCA cycle, and fatty acid oxidation are essential for nearly all cell types. Systemic inhibition may cause unacceptable toxicity, as demonstrated by 2DG. Broad mTOR inhibition with rapamycin impairs wound healing and increases infection risk. Similarly, the CANTOS trial revealed a small but significant increase in fatal infections with canakinumab, highlighting the dual-edged nature of anti-inflammatory therapy. Even seemingly safe interventions such as SGLT2 inhibitors carry risks of euglycemic diabetic ketoacidosis and genital infections, while colchicine can cause gastrointestinal intolerance and myopathy at therapeutic doses. These examples show a recurring theme: targeting immunometabolic pathways inevitably perturbs homeostatic immune function, and the therapeutic window for each strategy must be carefully defined through rigorous clinical testing rather than extrapolated from preclinical data.

To overcome these limitations, nanoparticle-based drug delivery systems have emerged as a transformative strategy for achieving cell-type specificity. Liposomal formulations, polymeric nanoparticles, and macrophage-targeted nanocarriers can deliver metabolic modulators preferentially to inflammatory macrophages or activated T cells while sparing quiescent cells. In atherosclerosis, nanoparticles functionalized with collagen IV-targeting peptides or dextran sulfate accumulate in atherosclerotic plaques, enabling localized delivery of anti-inflammatory payloads including NLRP3 inhibitors, itaconate derivatives, and siRNA targeting glycolytic enzymes. Lipid nanoparticle-mRNA platforms, refined through COVID-19 vaccine development, are being adapted to deliver metabolic enzymes or immunomodulatory proteins to specific immune cell populations. These delivery systems reduce off-target effects and widen the therapeutic window for metabolic interventions (107–113).

Additional emerging approaches include CRISPR-based functional genomics screens to identify metabolic genes selectively required for pathogenic immune cell functions but dispensable for protective immunity, mRNA-based therapies to transiently express metabolic modulators in inflamed tissues, and artificial intelligence-driven target discovery integrating multi-omics data to predict safe and disease-specific metabolic nodes.

### 6.6 Targeting trained immunity

The recognition that trained immunity contributes to both protective immunity and metabolic disease pathogenesis has spurred therapeutic strategies to selectively modulate innate immune memory (35, 44, 114).

First, harnessing beneficial trained immunity: BCG vaccination is being explored for non-specific protection against respiratory infections in metabolically vulnerable populations. Several clinical trials are evaluating BCG revaccination for reducing infection severity in individuals with type 2 diabetes, with preliminary data suggesting reduced respiratory infection rates. Additionally, β-glucan-based adjuvants are under investigation for enhancing vaccine responses in immunocompromised hosts with metabolic comorbidities. The challenge lies in inducing protective trained immunity without triggering maladaptive inflammatory amplification (42, 43).

Second, reversing maladaptive trained immunity: Statins, beyond lipid-lowering, inhibit cholesterol synthesis and modulate mevalonate pathway-dependent epigenetic modifications. The mevalonate pathway generates isoprenoid intermediates required for GTPase prenylation; by reducing these intermediates, statins attenuate the trained phenotype in monocytes from patients with atherosclerosis (39, 114). The BET protein inhibitor apabetalone (RVX-208) has shown promise in phase 3 trials for reducing cardiovascular events, potentially through disruption of bromodomain-dependent transcriptional memory at inflammatory gene loci (115, 116).

Third, targeting metabolic nodes that maintain the trained state: The Akt/mTOR/HIF-1α axis, glutaminolysis, and the mevalonate pathway represent druggable metabolic checkpoints for reversing pathological innate immune memory. Low-dose mTOR inhibitors, glutaminase inhibitors, and mevalonate pathway modulators are being evaluated in preclinical models of maladaptive trained immunity. Targeting specific histone modifications — particularly H3K4me3 and H3K27ac marks at inflammatory gene promoters — through histone methyltransferase or acetyltransferase inhibitors represents an emerging epigenetic strategy, though achieving gene-specific effects without global epigenetic disruption remains a formidable challenge (14, 39, 117).

Fourth, dietary and lifestyle interventions: Intermittent fasting may reset trained immunity by promoting autophagy and metabolic quiescence in hematopoietic progenitors. β-hydroxybutyrate-mediated NLRP3 inhibition and SIRT1 activation during fasting may contribute to erasing maladaptive epigenetic marks. The ketogenic diet, by shifting systemic fuel preference toward ketone bodies, may similarly attenuate trained immunity-dependent inflammation (101–103, 118).

The central challenge — and opportunity — lies in selectively reversing pathological trained immunity while preserving protective innate immune memory. Single-cell epigenomic and metabolomic profiling of trained immune cells in longitudinal human cohorts are likely to be essential for identifying molecular signatures that distinguish protective from maladaptive training. Integration of these signatures with clinical outcomes may enable precision immunometabolic therapy tailored to individual trained immunity profiles.

**Figure 5:**
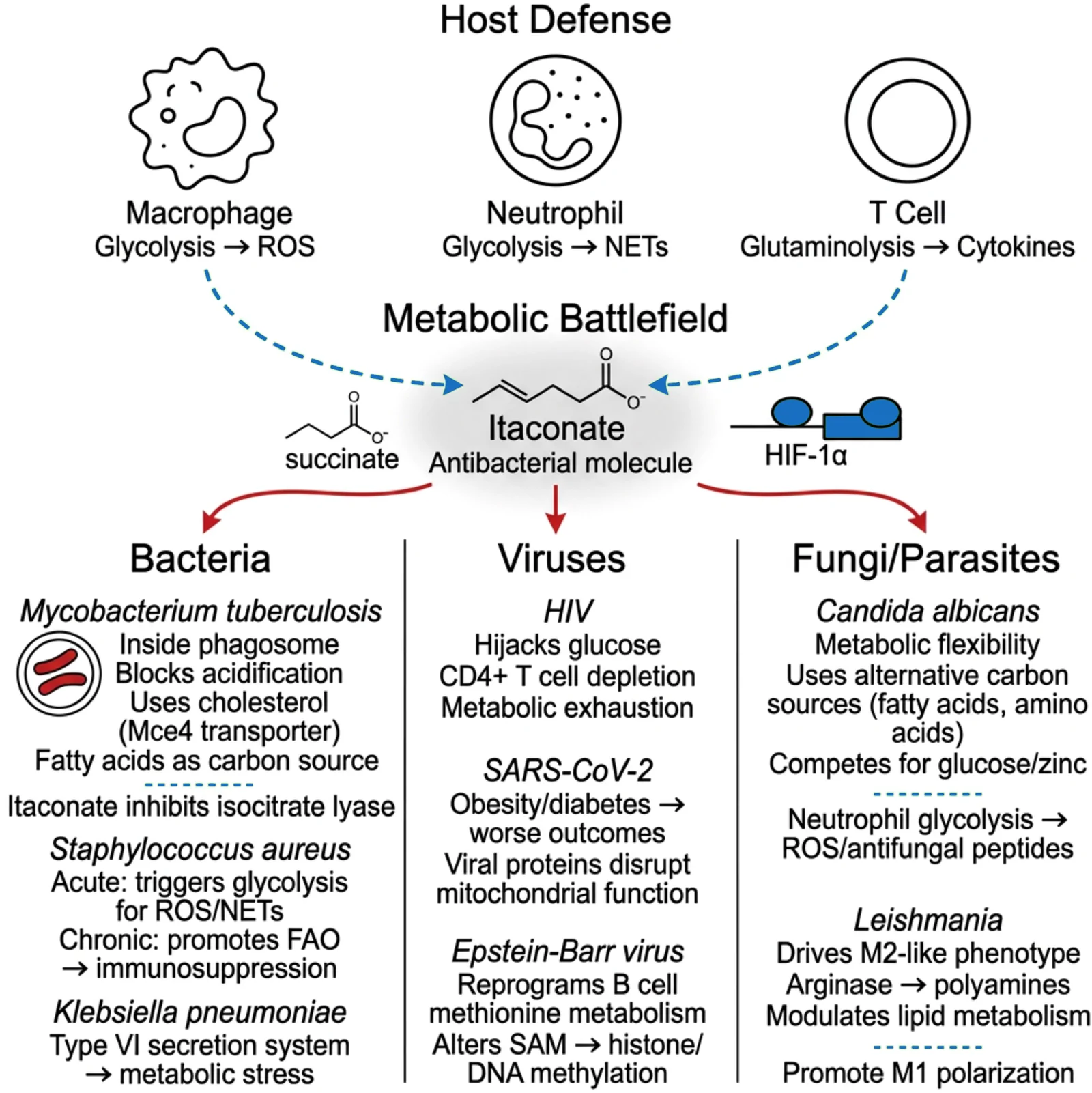
Therapeutic strategies targeting immune metabolism. Spider-web diagram with five spokes representing therapeutic modalities: (1) metabolic pathway modulators (metformin, SGLT2 inhibitors); (2) immunometabolic regulators (4-octyl-itaconate, natural products); (3) nutritional and lifestyle interventions (intermittent fasting, Mediterranean diet, β-hydroxybutyrate); (4) microbiota-targeted approaches (probiotics, fecal microbiota transplantation); and (5) emerging strategies (CRISPR-based editing, mRNA therapeutics, nanoparticle delivery, AI-driven drug discovery). A safety considerations box addresses risk-benefit balance and long-term metabolic consequences. Created with BioRender.com.

The major therapeutic strategies targeting immune metabolism, along with safety considerations, are summarized in **Figure 5**.

## 7 Challenges and future directions

Despite substantial progress, significant challenges remain. The complexity of *in vivo* metabolic interaction networks presents a fundamental obstacle: *in vitro* studies cannot fully replicate the heterogeneous, dynamic microenvironments within tissues, where extensive intercellular metabolite exchange occurs. Decoding metabolic dialogues between immune cells and neighboring cells in different tissues and disease stages is crucial for understanding overall pathophysiology (119).

Achieving specificity and safety in therapeutic targeting remains a central challenge. Core metabolic pathways are used by almost all cells, making it difficult to design drugs that precisely target specific immune cell subsets without disrupting normal cellular metabolism. Celltypespecific delivery systems, local administration routes, and intermittent dosing regimens may help address this challenge, but more selective targets are needed. Most clinical trials of immunometabolic interventions have been conducted in cardiovascular or metabolic disease populations, with infectious disease outcomes assessed only secondarily. Dedicated trials evaluating immunometabolic therapies for infection prevention or treatment — particularly in metabolically compromised hosts — are largely absent from the current literature.

Species differences between mice and humans in immune systems, metabolic rates, and lifespans hinder translation. Increased use of humanized mouse models, human primary cells, organoids, and clinical cohorts is needed to bridge this gap.

Individual variation in immunometabolic profiles — shaped by age, sex, genetics, microbiota, dietary history, and prior infections — complicates one-size-fits-all therapies. Integrating multi-omics and clinical data to define immune-metabolic subtypes represents the path toward precision immunometabolic therapy.

## 8 Conclusion

Over the past decade, immunometabolism has progressed from phenomenological description to mechanistic elucidation and intervention exploration. Metabolism serves not merely as a power plant or parts warehouse for immune cells but also as a decision-maker and memory bank that shapes immune responses. In metabolic diseases, immune cell metabolic reprogramming drives chronic inflammation and tissue dysfunction. In infectious diseases, the competition for metabolic resources between host and pathogen determines infection outcomes and shapes the evolution of both host defense and pathogen virulence strategies. Trained immunity provides a mechanistic basis for long-lasting innate immune memory with important implications for vaccine development and inflammatory disease (1–3).

These fundamental insights are now translating into therapeutic strategies. From repurposed drugs such as metformin and SGLT2 inhibitors to novel metabolic modulators such as itaconate derivatives, from nutritional and lifestyle interventions to microbiome-targeted therapies, targeting immunometabolism offers an integrated framework to address both metabolic and infectious diseases (90–92).

New technologies will continue to advance the field. Single-cell spatial multi-omics, live imaging, organoids, and artificial intelligence will provide higher resolution, better real-time dynamics, and greater integration of immunometabolic data. Technical advances in single-cell metabolomics — particularly improvements in temporal resolution and the development of spatial metabolomics for tissue sections — promise to reveal metabolite flux in intact tissue microenvironments. Although challenges in specificity, translation, and personalization remain, precision strategies centered on immunometabolism are likely to occupy a major place in future medicine, opening new paths to understand and treat complex diseases (120).
